# Chromatographic Method for Determining 6‑Mercaptopurine in Skin Permeation Assays for Assessing Cutaneous Exposure Risk

**DOI:** 10.1021/acsomega.5c04686

**Published:** 2025-07-16

**Authors:** Gabriel S. Oliveira, Ana Luiza Lima, Idejan P. Gross, Livia Sa-Barreto, Patrícia Medeiros-Souza, Tais Gratieri, Guilherme M. Gelfuso, Marcilio Cunha-Filho

**Affiliations:** † Laboratory of Food Drugs, and Cosmetics (LTMAC) 505616 University of Brasilia, Brasília, DF 70910-900, Brazil; ‡ Faculty of Ceilandia, University of Brasilia (UnB), Brasília, DF 72220-900, Brazil; § School of Health Sciences, University of Brasilia (UnB), Brasília, DF 72220-900, Brazil

## Abstract

6-Mercaptopurine monohydrate (6-MP) is a drug often used in leukemia chemotherapy, but limited information is available about its cutaneous absorption, which raises concerns about occupational risks for healthcare professionals and caregivers. This study aimed to validate an analytical method using high-performance liquid chromatography (HPLC) for quantifying 6-MP in the skin, allowing the assessment of its cutaneous permeation. The validation of the proposed method demonstrated high selectivity against skin interferents, linearity (*R* = 0.999), precision, and sensitivity, with detection and quantification limits of 0.13 μg/mL and 0.39 μg/mL, respectively. The developed extraction procedure allowed reproducible drug recovery from the stratum corneum and remaining skin, minimizing the impact of enzymatic metabolism in these biological matrices. Using the developed method, a preliminary skin permeation test on porcine skin showed that 6-MP is retained mainly in the remaining skin, accumulating approximately 2% of the total dose applied within 30 min of testing, suggesting a high risk of dermal exposure. The results reinforce the need for preventive measures, such as personal protective equipment and strict handling protocols, to minimize drug exposure. Thus, the method described constitutes an essential tool in assessing the risk of topical contamination of 6-MP and can be used in long-term studies of skin permeation of this drug, contributing to the safety of health professionals, caregivers, and patients.

## Graphical abstract

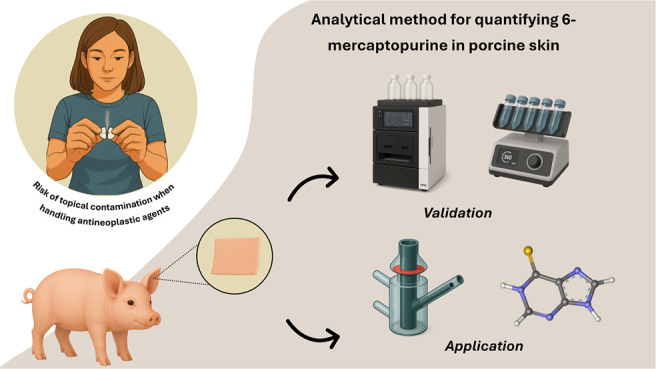

## 1 Introduction

6-mercaptopurine monohydrate (6-MP) is a drug widely used to treat acute lymphocytic leukemia and immunosuppressive therapies for inflammatory bowel diseases.^1–3^ As a purine analog, 6-MP requires metabolic conversion to exert its therapeutic effects, converted into 6-thioguanine nucleotides, which inhibit DNA and RNA synthesis and reduce cell proliferation.^4–6^

The metabolism of 6-MP occurs mainly through the enzymes hypoxanthine-guanine phosphoribosyl transferase, thiopurine S-methyltransferase, and xanthine oxidase, resulting in the formation of several active and inactive metabolites.^4,5,7^ Although the systemic pharmacokinetics of 6-MP are well characterized, as far as we know, there are no studies on its percutaneous absorption and distribution through the skin. The lack of information compromises the assessment of risks associated with occupational exposure and hampers the development of guidelines to minimize such potential risks.

The possibility of cutaneous absorption of 6-MP raises concerns about the safety of caregivers and healthcare professionals who come into contact with the drug during the administration and dispensing of its dosage forms.^8,9^ Routine handling may result in the involuntary absorption of the drug, potentially leading to serious adverse effects.^10^ In fact, cases of severe toxicity are common in patients undergoing treatment with 6-MP.^11^ However, it is unknown whether healthcare professionals and caregivers who handle the drug may suffer similar effects due to chronic dermal exposure. This reinforces the need to evaluate its skin permeation and establish safety guidelines to minimize potential risks.

The adverse effects of 6-MP include neutropenia, which increases susceptibility to infections, and hepatotoxicity due to the accumulation of toxic metabolites.^12,13^ In addition, severe dermatological reactions are reported, such as painful desquamation, cheilitis, hemorrhagic blisters, and perianal ulcers, especially in pediatric patients undergoing maintenance therapy.^11^ Such effects highlight the need to better understand its interaction with the skin and the potential risks of unintentional exposure.

In view of this scenario, quantifying 6-MP in the skin and performing skin permeation tests are essential to assess its absorption and distribution in such an organ. Chromatographic methods, such as high-performance liquid chromatography (HPLC), have already been used to quantify 6-MP in biological matrices, such as blood and urine, but there are still no validated methods for its determination in skin.^14,15^ Developing a robust analytical method will enable the investigation of 6-MP permeation through the skin, aiding in assessing occupational risks and establishing appropriate safety measures.

Therefore, this study aimed to develop and validate an analytical method for 6-MP quantification in the different layers of the skin using porcine skin as a model. Moreover, the utility of the developed method has been put to the test in a permeation assay to assess for the first time the potential risk of the drug’s cutaneous exposure.

## 2 Material and Methods

### 2.1 Materials

6-MP used as the primary standard (98%; lot MKCQ4491; water solubility = 6.9 mg/mL; log *p* = 0.01)^16,17^ was purchased from Sigma-Aldrich (St. Louis, MO, USA). 6-MP tablet 50 mg (Purinethol; lot 401773) was purchased from Excella GmbH and Co. KG. (Feucht, Bavaria, Germany). Chromatographic grade acetonitrile was obtained from J.T. Baker (Philipsburg, PA, USA). The water used in all experiments was ultrapure water obtained from Millipore (Illkirch Graffenstaden, France). Scotch 845 book tape (3M, St. Paul, MN, USA) was used for the tape stripping procedure on the skin. Porcine ears’ skin was obtained from a local slaughterhouse (Via Carnes, Formosa, Brazil).

### 2.2 Instrumentation and Analytical Conditions

Drug quantification was performed by HPLC in an LC-20AT equipment (Shimadzu, Kyoto, Japan) equipped with a DAD detector (model SPD-M20A), a degasser (DGU-20A3), a column oven (model CTO20AS), and an automatic sample injector (model SIL-20AD). Data acquisition and chromatogram plots were performed using Shimadzu LC software (LabSolutions, version 5.99, Kyoto, Japan).

For chromatographic separation, a C18 reversed-phase column 25 cm × 4.6 mm × 5 μm (Phenomenex, Torrance, CA, USA) was used as the stationary phase. The mobile phase was composed of acetonitrile and water in a 95:5 ratio (v/v) eluted isocratically at a flow rate of 1.0 mL/min. The injection volume of samples was 20 μL, and the column oven was maintained at 25 °C. Detection was performed at 327 nm, representing the maximum absorption wavelength in the UV spectrum for 6-MP.

Stock solutions of 6-MP (100 μg/mL) were prepared by dissolving 2.5 mg of the primary standard drug in 25 mL of acetonitrile with the aid of an ultrasonic bath. Then, dilutions were performed in acetonitrile. The performance of chromatographic analysis was carried out by calculating the theoretical plate numbers and the tailing factor with the aid of the Shimadzu LC software.

### 2.3 Preparation of Extracts from Skin Layers

The porcine fragments were obtained immediately after slaughter, before the scalping procedure, and transported to the laboratory under refrigeration. To separate the skin layers, the tape stripping technique was performed, allowing to obtain the stratum corneum and the remaining skin^18^ (Figure 1).

**Figure 1:**
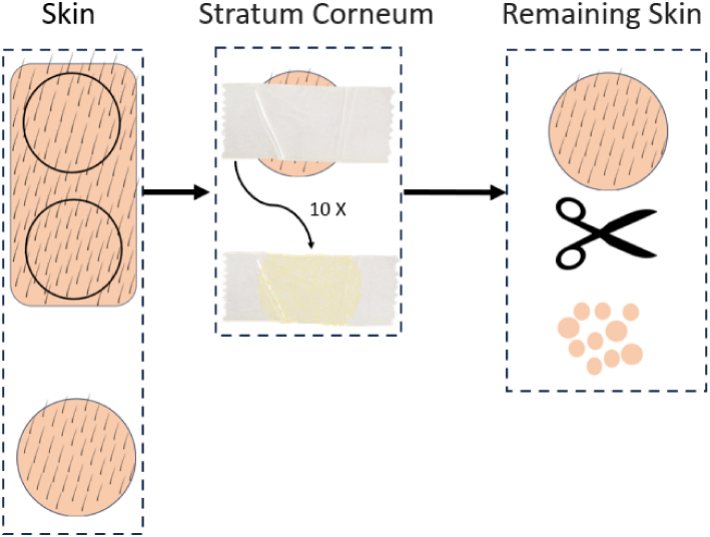
Scheme of the tape stripping technique with adhesive tape, allowing the obtaining of porcine layers skin separation, i.e., stratum corneum and remaining skin.

Briefly, three skin fragments, each with an area of 2 cm^2^, were removed from the porcine ears and fixed to a support, with the stratum corneum facing upward. Then, ten adhesive tapes were applied successively to remove the stratum corneum. The remaining skin was then cut into small pieces. Each of the separated skin layers was transferred to individual Falcon tubes, to which 5 mL of acetonitrile was added to obtain the extracts of the different layers used as biological interferents in the method validation. The samples were shaken for 48 h at room temperature using a rotary laboratory shaker (Multi Bio RS-24, Biosan, Riga, Latvia) and then filtered through hydrophilic polytetrafluoroethylene membranes with a pore size of 0.45 μm and a diameter of 25 mm.

### 2.4 Validation of the HPLC Analytical Method

In this study, the analytical HPLC method was developed and validated to quantify 6-MP in the different layers of the skin. The validation analyzed the parameters of selectivity, linearity, limit of detection (LOD) and quantification (LOQ), precision, and accuracy, following the guidelines of the International Conference on Harmonization (ICH) Q2 (R2).^19^

#### 2.4.1 Selectivity

A solution of 6-MP in acetonitrile was prepared at the nominal concentration of 5 μg/mL. Samples were tested in the presence or absence of the different skin interferents (stratum corneum and remaining skin, as described in Section 2.3) to evaluate whether the method can quantify the drug unequivocally and distinguish it from the interferents.^18,20^ The test was conducted in triplicate for each contaminant.

#### 2.4.2 Accuracy

Accuracy was assessed based on the recovery percentage of known amounts of 6-MP added to each separated skin layer. After separating the stratum corneum from the remaining skin following the tape stripping procedure (Section 2.3), known volumes of drug solutions in acetonitrile were added to Falcon tubes containing the respective skin fractions and left until completely dry. Next, 5 mL of the extraction solvent was added to the doped skin layers in order to achieve a final concentration of 10 μg mL^–1^ of 6-MP in each sample. Different solvents (acetonitrile, isopropanol, and dimethyl sulfoxide) and extraction times (48 and 72 h) were tested to identify the most efficient extraction method. Subsequently, the contents of each vial were filtered using 0.45 μm membranes and quantified according to the proposed method.

Drug recovery was calculated by dividing the concentration of 6-MP extracted from the skin layers (C_e_) by the theoretical concentration initially added (C_t_), according to eq 1.

1 $$\mathrm{Drug recovery}\;\left(\%\right)=\frac{C_{e}}{C_{t}}\times 100$$

#### 2.4.3 Linearity

The calibration curve was obtained using seven dilutions in acetonitrile at different concentrations (0.50, 1.0, 2.5, 5.0, 10.0, 15.0, and 20.0 μg mL^– 1^) prepared from three independent stock solutions of 6-MP. Statistical analysis of the data was performed using least-squares linear regression. Response factors were determined by the ratio between the chromatographic peak area and the analyte concentration. The test of significance of the angular coefficient and the test of proportionality were evaluated by the Student-*t* test (α = 0.05). The residues were calculated based on the difference between theoretical and experimental values. The normality of residues was checked by one-way analysis of variance (ANOVA) with a significance level of 0.05.^21^

#### 2.4.4 Limit of Detection (LD) and Quantification (LQ)

The determination of the limit of detection (LD) and the limit of quantification (LQ) of 6-MP was performed using the standard deviation (σ) and the slope (S) of the calibration curves, following eqs 2 and 3.^22^ These parameters are essential to assess the sensitivity of the analytical method, ensuring that the lowest detectable and quantifiable concentrations are accurately established. The LD represents the smallest amount of the drug that can be detected but not necessarily accurately quantified, while the LQ is the lowest concentration that can be measured with acceptable precision and accuracy within the assay conditions.

2 LD=3.3σ/S

3 LQ=10σ/S

#### 2.4.5 Precision

Precision was assessed at repeatability and intermediate precision levels. Repeatability (intra-assay) was determined by analyzing three concentrations of 6-MP (0.5, 5.0, and 20.0 μg/mL). Intermediate precision (interassay) was verified using solutions prepared on different days and by different analysts. All conditions were performed in triplicate. Results were expressed as the coefficient of variation (CV) calculated by dividing the standard deviation (SD) by the mean concentration (C_m_), according to eq 4.

4 $$\mathrm{CV}\;\left(\%\right)=\frac{\mathrm{SD}}{C_{m}}\times 100$$

### 2.5 Skin Permeation Test

To evaluate the utility of the developed method, an in vitro skin permeation assay was performed using porcine ear skin to assess the potential for contamination of the chemotherapeutic 6-MP during the handling of its tablet. Porcine skin is a suitable model for in vitro studies due to its close resemblance to human skin.^23^ The tests were conducted using a modified Saarbruecken permeation model.^24,25^ The porcine skin samples were fixed in the permeation model, with an exposed area of 1.7 cm^2^ and the stratum corneum facing upward. On the underside of the skin, a filter paper soaked in 0.01 mol L^– 1^ phosphate buffer solution (pH 7.4) was positioned, ensuring adequate skin hydration at the specified pH.

The commercial 6-MP tablet, containing 50 mg of the drug, was pulverized using a mortar and pestle. Each powdered tablet was applied to a skin sample previously moistened with 200 μL of water, as shown in Figure 2. The skin permeation experiments were conducted for 30 min in sextuplicate. After this period, the skin was lifted from the diffusion area, and excess drug was carefully removed from the surface using a spatula to gently scrape the skin. The tape stripping procedure was then applied to separate the skin layers and quantify the drug in each layer,^26,27^ as detailed in Section 2.3

**Figure 2:**
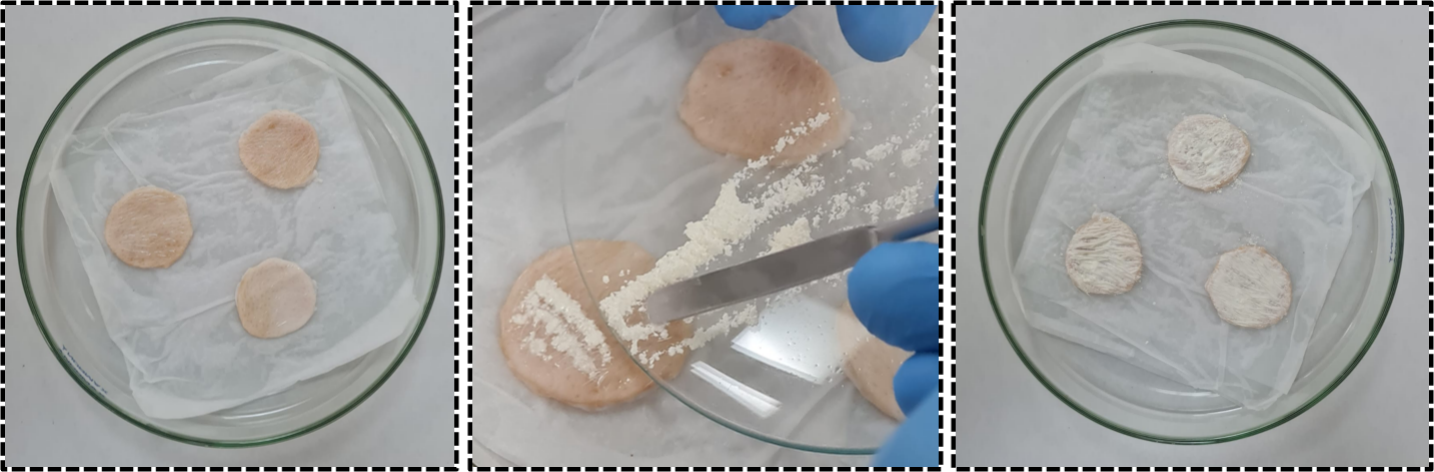
Modified Saarbruecken permeation model on porcine skin with 6-MP. Detail of the application of 6-MP powder tablets deposited on the skin.

## 3 Results and Discussion

The initial chromatographic conditions were optimized based on a previously published 6-MP determination method.^28^ The analysis conditions were modified until the final validated method described below was reached.

### 3.1 Selectivity

The method’s selectivity was confirmed by analyzing the chromatograms obtained for 6-MP in nominal concentration in the presence of the skin contaminants (Figure 3). Under the selected chromatographic conditions, no appreciable peaks related to the interferents were observed. Moreover, the presence of skin interferents in the analytical sample did not modify the chromatographic performance parameters of the analyte, evidencing that the components of the skin matrix did not compromise the quantification. In fact, the theoretical plate numbers above 2.000 indicated efficient elution of the analyte in the presence of contaminants, and the tailing factors were within the ideal range of 0.8–1.8, proving the symmetry of the analyte peak in all the analysis conditions evaluated.

**Figure 3:**
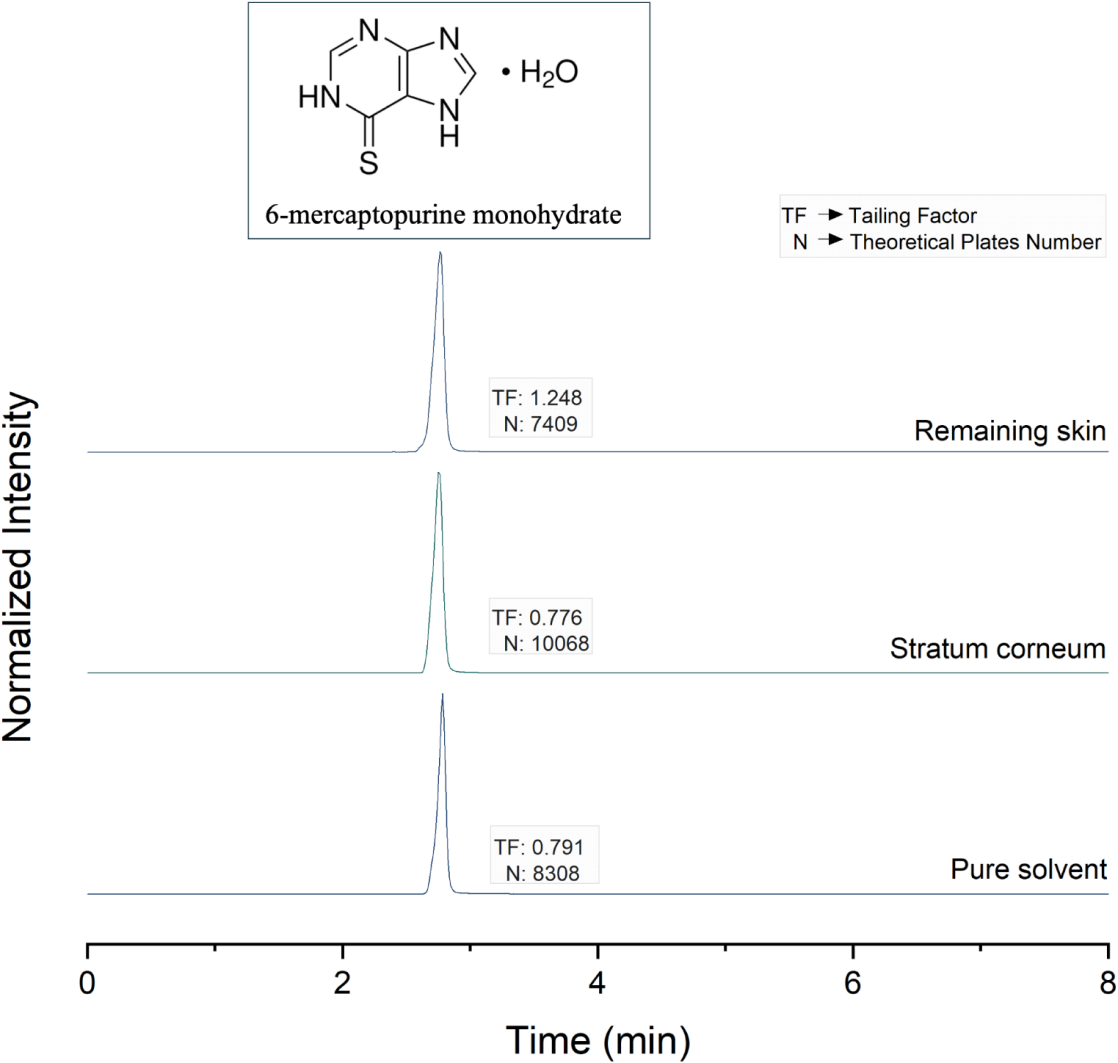
Chromatograms of 6-MP in acetonitrile in nominal concentration of 5 μg/mL containing different interferents from the skin layers (stratum corneum and remaining skin). 6-MP molecular structure is exhibited.

### 3.2 Accuracy

In bioanalytical methods, drug recovery from biological matrices is usually challenging. Indeed, it is often necessary to resort to more aggressive extraction procedures using, for example, surfactants, ultrasound, or high temperatures to remove the analyte from the biological matrices, which can, in turn, compromise their chemical integrity or elute too many interferents that make unfeasible the analysis.^18,27^

The development of the 6-MP recovery method from each skin layer was carried out using different extraction times and solvents in which the drug is highly soluble.^29–31^ The results presented in Table 1 demonstrate that acetonitrile was the most efficient solvent after 48 h of extraction, recovering 82.7 ± 2.2% of the 6-MP from the stratum corneum. Isopropanol, in turn, did not allow the quantification of 6-MP under any condition tested, and DMSO showed significantly lower recovery in all layers, with maximum values of 22.0 ± 1.7% in stratum corneum and 8.5 ± 5.1% in remaining skin.

**Table 1:** 6-MP Recovery Tests in Different Solvents and Extraction Times^a^

		6-MP recovery (%)	
Solvent	Extraction time (h)	stratum corneum	remaining skin
Acetonitrile	48	82.7 ± 2.2	31.5 ± 9.2
	72	79.1 ± 2.7	8.5 ± 7.4
Isopropanol	48	n.d.	n.d.
	72	n.d.	n.d.
DMSO	48	22.0 ± 1.7	8.5 ± 5.1
	72	8.0 ± 0.7	7.9 ± 3.3

a n.d = not determined.

Notably, the tests showed that prolonged extraction times reduced drug recovery. At 72 h, recovery in acetonitrile fell to 79.1 ± 2.7% in stratum corneum and to only 8.5 ± 7.4% in remaining skin, suggesting degradation of the analyte. In fact, as a prodrug, 6-MP can be metabolized by xanthine oxidase, an enzyme responsible for its hepatic inactivation and also present in the skin, converting it into thiouric acid, an inactive metabolite not quantifiable by the proposed method.^32^

Therefore, extraction using acetonitrile for 48 h was higher than 80% in the stratum corneum proved adequate for the analytical purposes. In the case of the remaining skin samples, we observed a result reproducible enough to apply a correction factor.^33^

### 3.3 Linearity

The linear regression equation obtained for the 6-MP analytical curve was determined as y = 119336x–20745, where y represents the detector response and x corresponds to the analyte concentration. The ANOVA confirmed the significance of the curves’ linearity, the variances’ homogeneity, and the residues’ normality. The correlation coefficient (r) calculated was 0.999, fulfilling the minimum recommended and indicating an excellent fit of the experimental concentrations to the regression. The analytical curve’s slope differed from zero, and the residues were randomly distributed without tendency.^34^

### 3.4 LD and LQ

The LD and LQ limits of the method developed for the analysis of 6-MP were determined as 0.13 μg/mL and 0.39 μg/mL, respectively. These values indicate the technique’s high sensitivity, allowing the detection of minimal amounts of the drug in the sample. Indeed, considering the permeation conditions used in this study, in which 50 mg of 6-MP was applied to the skin, the LQ would be sensitive enough to quantify at least 0.02% of this dose in any skin fractions. Thus, the results reinforce the method’s suitability in identifying and quantifying minute quantities of the analyte in the biological matrix of the skin, even in short-time permeation tests.

### 3.5 Precision

The method repeatability assessment was performed using three different concentrations of 6-MP, and the results obtained showed that the CV values were consistently below 2%, as shown in Table 2. The coefficient of variation below 2% suggests that the method maintains a stable and reproducible performance level within the range of concentrations tested, demonstrating its reliability for 6-MP measurements under routine conditions.

**Table 2:** Accuracy of the HPLC-UV Method for the Determination of 6-MP

Theoretical concentration (μg/mL)		Experimental concentration (μg/mL)		CV (%)
Repeatability	0.50	0.65 ± 0.00		0.29
	5.00	5.29 ± 0.07		1.25
	20.00	21.77 ± 0.14		0.63
Intermediate precision	Analyst 1	Day 1	Day 2	
	0.50	0.51 ± 0.00	0.58 ± 0.01	6.74
	5.00	5.32 ± 0.02	4.99 ± 0.12	3.79
	20.00	21.64 ± 0.28	19.97 ± 0.77	5.05
	Analyst 2	Day 1	Day 2	
	0.50	0.49 ± 0.01	0.54 ± 0.02	6.27
	5.00	5.02 ± 0.15	5.99 ± 0.21	10.04
	20.00	19.06 ± 0.23	18.24 ± 0.19	2.61

In addition, the method was also evaluated at the intermediate precision level, which involved different analysts on different days. The results of this evaluation confirmed that the method is robust, with CV consistently below the recommendations for a bioanalytical method (<15%) and capable of maintaining adequate performance regardless of variations related to different operators, reinforcing its applicability in different laboratory environments and ensuring the reliability of the quantifications performed.^35,36^

### 3.6 Skin Permeation Study

The skin permeation study was performed to evaluate the feasibility of using the proposed method to assess the potential risk of skin contamination during the handling and subdivision of commercial 6-MP tablets. The assay used powdered tablets simulating direct skin contact with the drug, resulting from accidental exposure. Specifically, in the case of 6-MP tablets, the practice of tablet subdivision to adjust doses is quite common, especially in the case of pediatric patients.^37^ The procedure of subdividing the tablets leads to their crumbling, resulting in direct skin exposure to the drug with the operator’s hands.^8,9^

The results presented in Figure 4 showed that even in a solid state and for a short period (30 min), a relevant amount of drug permeated the remaining skin. In fact, the dose permeated into deeper skin layers represents approximately 2% of the total dose in a tablet. Although the quantified 6-MP is in an inactive form, its retention in the skin raises concerns regarding occupational and unintentional exposure, especially due to its high toxicity.

**Figure 4:**
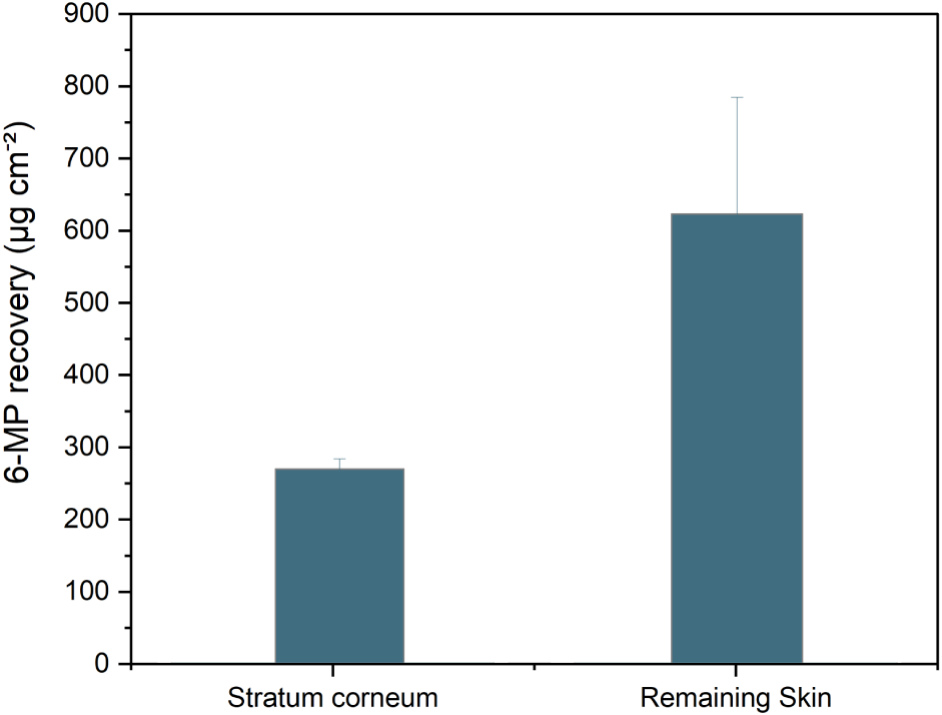
Amount of 6-MP quantified in the different layers of the skin (stratum corneum and remaining skin) after the skin permeation test. Error bars represent the standard deviation of six independent experiments (*n* = 6).

Studies indicate that the transdermal route may allow systemic absorption of drugs, even for compounds with physicochemical characteristics unfavorable for skin permeation.^38^ Furthermore, research with azathioprine, a prodrug of 6-MP, has shown that its absorption through the skin can lead to metabolic conversion to active 6-MP.^39^ Thus, contact with the skin, even if it results in minimal absorption, represents a potential risk since the drug may remain on the skin surface and be inadvertently transferred to mucous membranes or other areas of the body.

Improper handling of the drug may result in prolonged and cumulative exposure, increasing the risk of local or systemic adverse effects. In addition, enzymatic conversion in the skin may amplify these risks, given the active form’s ability to cause cellular damage.

In fact, despite the short permeation period (30 min), the amount that penetrated the deepest layers of the skin is twice as great as the amount retained in the stratum corneum, which indicates the high permeability of this molecule and the potential risk of adverse effects. Therefore, the results reinforce the need to adopt strict preventive measures, such as personal protective equipment and hygiene protocols, to minimize risks to patients, caregivers, and healthcare professionals.

## 4 Conclusions

The HPLC analytical method was developed and validated for the determination of 6-MP in skin permeation assays, which proved to be selective for biological contaminants of the skin. Furthermore, the method demonstrated linearity, precision, and sensitivity for quantifying the analyte in minimal quantities. Finally, the extraction procedure allowed for reproducible drug recovery from both the most superficial and deepest layers of the skin, minimizing the impact of drug metabolization in the skin. The skin permeation assay using the developed method indicated that the chemotherapy can be retained in the skin, mainly in the deepest layers of the epidermis, suggesting a high risk of occupational and unintentional exposure. These findings highlight the importance of implementing preventive measures when handling 6-MP, such as wearing gloves, avoiding splitting tablets without appropriate containment, and strictly adhering to institutional safety protocols. Thus, the method described is useful in assessing the risk of topical contamination of 6-MP and can be used in studies of skin permeation of this chemotherapy drug, contributing to the safety of health professionals, caregivers, and patients.
